# Experiencing ‘pathologized presence and normalized absence’; understanding health related experiences and access to health care among Iraqi and Somali asylum seekers, refugees and persons without legal status

**DOI:** 10.1186/s12889-015-2279-z

**Published:** 2015-09-19

**Authors:** Mei Lan Fang, Judith Sixsmith, Rebecca Lawthom, Ilana Mountian, Afifa Shahrin

**Affiliations:** Gerontology Research Centre, Simon Fraser University, 2800 - 515 West Hastings Street, Vancouver, BC V6B 5K3 Canada; School of Public Policy, Simon Fraser University, Vancouver, Canada; Institute of Health and Wellbeing, University of Northampton, Northampton, England; Research Institute for Health and Social Change, Manchester Metropolitan University, Manchester, England; Instituto de Psicologia, Universidade de Sao Paulo, Sao Paulo, Brazil

**Keywords:** Minoritization processes, Othering, Asylum seekers, Refugees, Persons without legal status, Experiences of health and wellbeing, Health care access, Qualitative methods

## Abstract

**Background:**

Asylum seekers, refugees and persons without legal status have been reported to experience a range of difficulties when accessing public services and supports in the UK. While research has identified health care barriers to equitable access such as language difficulties, it has not considered the broader social contexts of marginalization experienced through the dynamics of ‘othering’. The current study explores health and health care experiences of Somali and Iraqi asylum seekers, refugees and persons without legal status, highlighting ‘minoritization’ processes and the ‘pathologization’ of difference as analytical lenses to understand the multiple layers of oppression that contribute to health inequities.

**Methods:**

For the study, qualitative methods were used to document the lived experiences of asylum seekers, refugees and persons without legal status. Thirty-five in-depth interviews and five focus groups were used to explore personal accounts, reveal shared understandings and enable social, cognitive and emotional understandings of on-going health problems and challenges when seeking treatment and care. A participatory framework was undertaken which inspired collaborative workings with local organizations that worked directly with asylum seekers, refugees and persons without legal status.

**Results:**

The analysis revealed four key themes: 1) pre-departure histories and post-arrival challenges; 2) legal status; 3) health knowledges and procedural barriers as well as 4) language and cultural competence. Confidentiality, trust, wait times and short doctor-patient consultations were emphasized as being insufficient for culturally specific communications and often translating into inadequate treatment and care. Barriers to accessing health care was associated with social disadvantage and restrictions of the broader welfare system suggesting that a re-evaluation of the asylum seeking process is required to improve the situation.

**Discussions:**

Macro- and micro-level intersections of accustomed societal beliefs, practices and norms, broad-levellegislation and policy decisions, and health care and social services delivery methods have affected the health and health care experiences of forced migrants that reside in the UK. Research highlights how ‘minoritization processes,’ influencing the intersections between social identities, can hinder access to and delivery of health and social services to vulnerable groups. Similar findings were reported here; and the most influential mechanism directly impacting health and access to health and social services was legal status.

**Conclusions:**

Equitable health care provision requires systemic change that incorporate understandings of marginalization, ‘othering’ processes and the intersections between the past histories and everyday realities of asylum seekers, refugees and persons without legal status.

## Background

Asylum seekers, refugees and persons without legal status (i.e. individuals situated between legal positions who find themselves without legal status and are awaiting deportation) in the UK can experience huge difficulties acquiring health care; despite the National Health Service principle of care being freely available at the point of access [1]. Such difficulties arise due to particular pre-departure histories and post-arrival challenges that create the conditions for the development and prolongation of various physical and mental health outcomes such as schizophrenia, suicidal ideation/attempts, anxiety disorder, depression and post-traumatic stress disorder; all of which pertain to experiences of war and challenges associated with resettlement [2–4].

For asylum seekers, refugees and individuals without legal status in the UK, access to appropriate health and social care supports and services can be challenging and procedurally onerous creating a public health problem in ensuring equal access to health care [5]. Difficulties in accessing health care relate to a number of barriers such as: excessive paper work; limited number of trained staff to provide culturally competent health care; limited number of interpreters trained to work in a medical setting; and lastly limited health status information of people seeking asylum [6]. Meanwhile, the nature and impact of such difficulties on the health of asylum seekers, refugees and persons without legal status are scarce or are unknown. Research by Mountian [7] has indicated that forced migrants when accessing health care experience a range of barriers that stem from: lack of information on how to access services; types of services available to them; language barriers; lack of cultural competency; fear of persecution; as well as systemic issues associated with being ‘status less’ (i.e. the transitioning process from an asylum seeker to a refugee).

At present, there is limited quantitative and even scarcer qualitative data on the health experiences of asylum seekers, refugees and persons without legal status in the UK. Aggregated knowledge (usually collected through questionnaires) tends to homogenize and simplify the complexity of the diverse experiences of asylum seekers, refugees and individuals without legal status. Inattentiveness to specific cultural and religious nuances and contexts between and within groups may further marginalize refugees and asylum seeker in the British health care system. New knowledge for the development of effective services and programs that tailor to the specific needs of these groups is required through more in-depth explorations into their everyday lives.

This research takes into account such complexities by exploring health experiences and access to health care of asylum seekers, refugees as well as those that do not fit neatly into any immigration classification. We examined qualitative research [8] that focused on socio-cultural understandings of Somali and Iraqi asylum seekers, refugees and persons without legal status living in Manchester (UK), identifying multiple forms of oppression to provide solid context to situate the current research.

Our analysis conceptualizes the data by theoretically elaborating on the beliefs and practices associated with ‘normalized absence, pathologized presence,’ an idea and phrase coined by Phoenix [9]. Broadly, it can be interpreted as the social exclusion of a particular group or groups based on negative stereotypes and assumptions [9]. For example, Somali and Iraqi refugees and asylum seekers are considered *invisible* in everyday British society. Their visibility becomes apparent only when their presence is problematized as a hindrance or burden imposed on British peoples. Historically, theoretical underpinnings of Phoenix’s couplet were based on the exclusion of black people in research studies because they were (and likely still are) labeled as being ‘exceptions to the norm, deviant or pathological’. The rationale behind this approach comes from the process of ‘othering’ or more precisely ‘minoritizing’ and the importance of examining how the position of the ‘other’ may inform research in this area [10–13]. Here, ‘othering’ is used to describe people that are socially situated outside the ‘boundaries of belonging [10, 11].’ Indeed, this research is timely given the current migration debates and the ‘othering’ of migrants seeking ingress across the EU [14, 15]. For example, Somali and Iraqi asylum seekers, refugees and persons without legal status living in the UK are consistently represented as a threat to the dominant notions of what is considered ‘community’ and ‘sovereignty’ [10, 11]. Subsequently, we posit that this concept can be applied to the health experiences of Somali and Iraqi asylum seekers, refugees and persons without legal status in the UK because of their social marginalization [16], disempowerment [17] within and exclusion from social systems [10].

‘Othering’ entails marginalization, disempowerment and social exclusion securing hegemonic identities by distancing and stigmatizing those that hold characteristics, which deviate from the status quo [10, 11]. This effectively creates a divide between ‘us’ and ‘them,’ which coincides with the concept of ‘normalized absence, pathologized presence,’ since the presence of the ‘other’ is often viewed as being intrusive whereas their absence is often preferred and widely accepted as being ‘the norm’ [9, 10]. Somali and Iraqi asylum seekers, refugees and individuals without legal status in the UK, are often stigmatized as being disingenuous, burdensome and a drain on resources [10] fitting the notion of ‘pathologized presence.’ Simultaneously, the process of ‘normalized absence’ often renders these individuals invisible in the health care system through the neglect of cultural and religious needs in health care policy. Providing an outlet to share personal experiences, in particular, challenges associated with external living conditions may help chip away at the socially ascribed ‘otherness’ associated with asylum seekers, refugees and those in precarious legal positions. Storytelling [18] is a mechanism that can be used to increase knowledge of those situated on the opposite side of the ‘other’ – individuals that criticize and/or lack understanding of the positions of forced migrants.

National and international research has emphasized the importance of addressing displaced persons’ experiences of war trauma [3, 4], political upheaval [19], persecution and torture [20], and challenges associated with resettlement [15, 21] and cross-cultural differences when developing and implementing health equity initiatives [22, 23]. There is a need to explore the broader determinants of health, including forced migration. Emphasis must be placed on addressing associated macro-level structural factors. For example, it has found that changes in UK legislation negatively affected asylum seekers’, refugees’, and status less persons’ health conditions through social exclusion, poverty, isolation and stigmatization [20, 24, 25].

Key mechanisms of ‘othering’ are influenced by differing social identities that concern gender, race, class, age, education, religion and social, political and cultural background [10]. In the case of Somali and Iraqi asylum seekers, refugees and persons without legal status in the UK, social constructions of the ‘other,’ generate modes of differentiation that fuel social stratification and creates social distance between individuals that require safety and refuge and persons from the host society. This paper aims to apply the social dynamics of ‘normalized absence, pathologized presence’ to the case of Somali and Iraqi asylum seekers, refugees and persons in precarious legal immigration positions residing in the UK and demonstrate how this is reinforced through the process of ‘othering.’ Experiential findings will reveal how multiple forms of oppression are facilitated through the process of ‘othering’ and influences the production and reproduction of inequitable health outcomes amongst these groups.

## Methods

Qualitative methods were used to identify key elements of the lived experiences of Somali and Iraqi asylum seekers, refugees and persons without legal status. The study focused on Somali and Iraqi participants, as both groups had recently became prominent in the Manchester Metropolitan area, with similar pre-arrival histories and post-arrival challenges concerning war-trauma and discrimination through the ‘othering’ process. To help facilitate and incorporate participant input throughout the research process, the research team liaised with a range of community-based and voluntary black and minority ethnic group organizations to help provide access to asylum seekers, refugees and persons without legal status and interview support. This local, knowledge-based dialogue enhanced ways of contacting the ‘seldom heard’ participants enabling them to have a voice in the project. ‘Seldom heard’ participants refer to people who are often intentionally or unintentionally neglected by researchers and public health workers.

A participatory framework was undertaken which involved collaborative workings with local organizations that worked directly with asylum seekers, refugees and persons without legal status. Furthermore, the inclusion of asylum seekers and refugees as co-researchers was facilitated throughout the research process. Here, four refugee community members were recruited, trained and supported to collect interview data and to engage in data interpretation in individual and two analytical workshops. Where appropriate skills and desire existed, co–researchers were also invited to take part in the report writing process. This was important because it allowed the researchers to interview individuals with no or minimal English speaking skills. A summary of findings was provided to key community-based organizations.

Five focus groups (*N* = 56 Somali, *N* = 10 Iraqi) were conducted to reveal shared understandings of health and access to health services and 35 in-depth interviews were conducted to explore personal experiences and enable social, cognitive and emotional understandings of on-going health problems as well as access to health care services. The focus groups were approximately 2 hours in duration and were stratified by gender and ethnicity as we adopted a Cultural Safety^1^ lens to ensure compatibility with cultural expectations. There were two Somali male focus groups (*N* = 30 with 1 service provider, *N* = 10); one Somali female group (*N* = 12 with 3 service providers); one Iraqi male group (*N* = 4) and one Iraqi female group (*N* = 6). Focus groups topics included experiences of accessing health services, cultural competency and safety within health care contexts and overall wellbeing.

The interview sample comprised 15 refugees, 12 asylum seekers and 8 participants that were receiving/applying for Section 4 support^2^ (at the time of the interview). Almost two-thirds of refugees and asylum seekers were Somali. However, among the eight people who were receiving/applying for Section 4 support, five were Iraqi. The sample was stratified according to nationality, gender, age, and time of migration. Among the interview sample, 20 (57 %) were males and 15 (43 %) were females. All participants had been living in the UK for several months to more than 10 years. Their age ranged between 21 and 74 years. Interviews lasted between 30 and 60 min and were conducted at venues chosen by participants. Where co-researchers were not available, interpreters were present where participants were not proficient in English.

Interviews were recorded, transcribed (translated into English where necessary by volunteers from community partner organizations), written into stories and where possible; authenticated by participants. Interviews were not subject to ‘translation and back translation’ due to the financial constraints of the project. A thematic analysis was conducted [26] exploring the experiences of health and access to health care in a population of Manchester’s Somali and Iraqi asylum seekers and refugees [8]. Analytical steps in the thematic analysis involved six key stages: familiarization (reading and rereading transcripts and notes and identifying initial ideas); code generation (systematic identification of interesting ideas); theme identification (collating codes into potential themes supported by relevant data); review (holistically checking themes against data and creating thematic map); labeling themes (refining specifics of themes and defining holistic analytical accounts in relation to research questions) and report writing [26]. Both principal project team members and participant researchers contributed to the analysis.

A combination of feminist theoretical concepts were used in this analysis to capture themes or mechanisms that facilitate the process ‘othering,’ specifically, ‘minoritizing,’ in order to demonstrate how the presence of Somali and Iraqi asylum seekers and refuges residing in the UK are pathologized and their absence normalized [11, 12]. Key interviews were analyzed within the socio-political, cultural and family contexts of a deprived urban area. As a part of the feminist methodological approach, the storytelling method was selected to enable researchers to understand the day-to-day activities, events and challenges experienced by refugees and asylum seekers in the UK [18]. More importantly, this method served to liberate and empower the research participants by providing opportunities for them to share their stories and voice their thoughts and opinions [18].

In order to protect participants’ anonymity, demographic and thick descriptive information has not been included in the quotations of this paper. The participants were provided informed consent prior to the interview, which ensured their anonymity and the right to withdraw from the research when they wanted. Where upsetting or difficult situations or emotions arose during data collection, the researcher gave the participant time to experience these and recover, they withdrew or continued with the interview as requested by the participant and offered service phone numbers (and if necessary, helped the participant contact service organizations to gain any support needed). Appropriate ethics approval was collected from the Ethics Committee at Manchester Metropolitan University, the National Heath Service and the National Research Ethics Service.

## Results

Our findings focused, firstly, on Somali and Iraqi asylum seeker and refugees’ experiences of health, secondly, how these experiences were shaped by difficulties accessing health care services and thirdly, how other post-arrival struggles impacted their health and well being. Broad-based themes revealed intersecting mechanisms that influenced the participants’ experiences of health and well-being, and depicted how they were operationalized within ‘othering;’ processes that reinforced fundamental ideals of ‘pathologized presence, normalized absence’ of asylum seekers and refugees. The four themes identified include: 1) pre-departure histories and post-arrival challenges, 2) legal status, 3) health knowledges and procedural barriers and 4) language and cultural competence.

### Theme 1: pre-departure histories and post-arrival challenges

Experiences of war, political upheaval, persecution and torture were revealed as some of the key reasons for relocating to the UK. Several participants indicated that pre-departure experiences affected their ability to build a new life in the UK. Consequently, issues of confidentiality and trust were prominent amongst participants in this study. Such issues translated into reluctance to visit health care services. Specifically, participants expressed concerns regarding trust in interpreters and health care professionals and the implications of misplaced trust for their community reputation and eligibility to remain in the UK. These were deeply rooted and framed in terms of past experiences of trauma and new experiences of exclusion whereby participants had felt bullied by powerful officials.

For example, one participant described going through the motions of ‘eating’ and ‘sleeping’ whilst not being able to forget traumatic experiences of the past:

I did not want to live because of what is gone. I was eating, sleeping only […] I decided to forget it all, but it came back to my mind […] I tried to forget it but I can’t. My history, my life is stamped into my heart.

Similarly, another participant revealed how post-war experiences culminated his will to live:

What happened to me was that my house was bombed and I woke up, you know, I woke up in the hospital in Somalia with no legs and part of my family […] I was 12 years old when I lost the legs. I tried to commit suicide several times because it was too hard on me.

Several participants spoke about the persecution they had experienced in their homelands as voiced by one Kurdish Iraqi participant. This individual was placed in a Kurdish prison in Iraq where his life was threatened. He was granted refuge in the UK and this saved him, however, he was forced to leave his family behind. Such experiences had impacted his ability to reclaim a new start in the UK:

When I came to the UK I was not happy. My family at home has problems. The life of my family is not good because of the terrorists in Iraq. They threaten them, to kill them the terrorists in Iraq always threatening. That is why I am not happy here. Always they are in his [relative’s] city.

Close encounters with death and experiences of persecution in war-torn countries, for many participants, resulted in long-term mental health implications.

I think many of them, they are sad people. They have problems with their lives. They are irritable people and are mentally thinking all the time about their futures. Psychologically I think they have are not good because they haven’t got any hopes with their lives, any sparkle, and they are afraid to be sent back to Iraq.’

Resettlement challenges also had impacted well being and these included: language barriers; loss of social and cultural capital; discrimination; racism; stigma as well as geographic, climate and food differences.

For example, participants shared stories that captured experiences of discrimination (i.e. name calling, stoning and/or being denied services on the based on how they are portrayed in the media as ‘untrustworthy,’ ‘dangerous,’ and ‘uncivilized’). One individual described being forced out of a lawyer’s office and stereotyped as being untrustworthy, ‘So I’ve not seen my family (in Somalia) because of him [lawyer] and when I went to him they chased me like they don‘t trust me, and said *bogus asylum, go away*.’ Another individual highlighted conversations and media reports, which positioned him in ways he felt were demeaning and discriminatory:

Many times I’m talking with a girl and the girl say “Where you come from?” and I say Iraq, and she says “What are you doing here?” and I say, I say just, “Asylum seeker” because I don’t want to say, be lying. She be laughing and she say “Is it possible to be friends with asylum seeker?” and I say, “Why?” When I went to England I hear about asylum seeker and I think bad things, bad things the government shouldn’t say, “These people are asylum seekers,” they should say “They are human.” To not make different between the people.

The analysis revealed several participants had experienced some form of discrimination in the UK and throughout Europe. Feelings of being ‘distraught’ were emphasized as individuals noted having escaped one type persecution only to be met by another more insidious form. This issue is systemic in nature, since forced migrants are prescribed titles such as ‘asylum seeker’ or ‘refugee,’ labels which are ultimately embedded with restrictions that engage ‘othering’ processes. Official labels signify not only difference, but also pejorative associations, and formally institute a range of exclusions from mainstream society. Consequently, if ‘othering’ is imposed from the top down via government legislation and policy, this shapes public perspectives and mainstreams exclusionary processes.

### Theme 2: legal status

Temporary ‘asylum’ status contributes to ongoing uncertainty, insecurity and the potential of forced return. In the UK, newly arrived asylum seekers often rely on a combination of social services and charitable or faith-based non-governmental organizations to assist in the process of resettlement. Services rendered by the government are, largely, only available to those with asylum. In the UK, asylum seekers are provided limited supports. These may include housing, health care and some financial means to help pay for food and other necessities.

Difficulties accessing health and social care stem from not having a stable home address. For example, without an address, GPs will not register patients. As well, individuals are not eligible to receive financial supports. Failed asylum seekers (individuals with applications that have been refused) and those classified under Section 4 are the extremely disadvantaged as they are ineligible for most public funds and services. The first passage contextualizes how having inadequate legal status restricted individuals from acquiring an NHS Medical Card resulting in refused medical attention from local GPs.

When I came I was sad all the time, can’t sleep at night. I talk about it. I ask if I can get help. If you don’t have card doctors can’t accept you, no one can, because you are a refugee, you don’t have the rights. Some people, you know, they know that. Maybe the problems is like that way.

A failed asylum seeker who revealed receiving good health care upon arrival recounted a similar story. Incidentally, once asylum status was refused, access to public resources was severely limited and medical aid denied:

The first one [GP at a hospital] I went to here, they look after me like really well and nicely. After I get refused [asylum] everything stopped. Nothing come good for me. I been [to see] a doctor and doctor say, “You are [failed] asylum seeker, I can’t help you.”

As specified, failed asylum seekers and individuals under Section 4 support have limited recourse to public funds and services. In many instances, barriers to accessing health care stem from stringent bureaucratic systems. For example, individuals who are refused asylum often have unstable housing situations. Without an address, persons without asylum are denied health care and treatment since residential information is required for GP registration:

I got refused. I didn’t have address so nobody is accepted. If you are refused you don’t have address, you know. When you go [to the doctors] they say “what is your address” and if you don’t have, they don’t see you.

The analysis revealed that participants were often unclear as to whether they were eligible for services provided by GPs, dentists, specialists and other supporting organizations. They were also unsure as to why such systems were set up in ways, which made them feel ‘othered’:

I want to see a specialist, because I have problems. First back pain, then knee pain and then problems with my life. And I have problems with my teeth, but they don’t give me appointment for the dentist. It is difficult because every time I need an interpreter. I didn’t find a dentist. I am destitute and don’t know what to do. I only have vouchers. One time I went to ASHA [Asylum Support Housing Advice] and they told me to see a solicitor to make a fresh claim. I cannot go back to Iraq because there life is danger.

Findings highlight that health services were often sought for problems other than those deemed medically related. GPs are frequently confronted with issues that are associated with well being. In the case of refugees and asylum seekers with challenging pasts, this is not an uncommon scenario nor is it an unreasonable request. It is important that health care providers are open to learning the stories of people with more complex needs. Failure to acknowledge the role of pre-departure histories and the importance of these in health trajectories sets conditions for experiences of ‘normalized absence’ as the person’s past history is absented from the health care process.

### Theme 3: health knowledges and procedural barriers

While asylum seekers and refugees have access to health services in theory, in practice, there are many difficulties. Failed asylum seekers and those under Section 4 support have limited legal recourse to health care provision. Additionally, they may also experience challenges that relate to: lack of familiarity with the UK health system; limited knowledge of the different health services that are available; processes and procedures to accessing health services; cross-cultural relational differences between doctors and patients; inconsistent advice from statutory and voluntary sources; difficulties navigating bureaucratic systems; mistrust of authority; language barriers and confidentiality. These are contextualized in the next passages.

One of the most frequently shared health seeking experiences involved ‘waiting.’ Differences in cultural experiences regarding immediate access to GPs contrasted poorly with waiting times and appointment systems:

In my homeland we went to the GP and be seen the same day, but here you have to make an appointment and there might be a waiting list. Sometimes they give you more than 7 days. Sometimes my condition may improve so is not necessary to make an appointment for 7 days then at that time things may change. I think this can make it difficult to go.

The problem with a long ‘wait and see’ period is that it may prevent individuals with more serious illnesses from acquiring medical attention at an early point in the disease process.

Another key issue identified concerns lack of knowledge on how to access primary health care services. Not knowing who to ask or where to go for health care resulted in frustration and desperation, and prompted help seeking from inappropriate sources:

Tell me you go see GP. Go in GP, come back again in GP I tell “Please, I go hospital they tell me, doctor, no see here, see GP, see you”. I see woman sit down in reception, I tell “Please, where is my doctor?” Me have this problem, asthma, headache, heart pain. “No, sorry. Mr. H is busy.” I tell, “Thank you very much.“ Me come back again, tomorrow me come back again.” Night me no sleep for then, no sleep. I go in tomorrow still the same “What, no busy”. I tell GP “Please me have problem.” “No, busy”. After me go out I see police, I tell police “Please come see my problem”. Police tell me “See GP, ask manager”. Me come back again in GP I tell, “Please, where is the manager? Where sit down?” Manager holiday. Manager on holiday.

With limited knowledge on how to effectively navigate the health care system, the individual’s presence within the system was pathologized. In the case of persons with Section 4, access to health care can be a substantially more onerous process, as they may not have the financial means to get to GP appointments. This is emphasized in the next passage, highlighting the ways in which health care professionals underestimate the problems faced by asylum seekers with little or no income:

My doctor is closing door, nurse tell me, “Your doctor busy. You go back, tomorrow come here”. I tell, please understand, no have money. This [bus pass] finished. With what coming here? People understand my problem, just no help.

Another frequent topic concerns short consultations. Several participants felt that GP consultations were too hastened to encourage full and honest assessments of individual health statuses. It was revealed that on average their time spent with GPs would last between 5 and 10 min. This was particular a problem when discussing sexual health or other topics that are more sensitive in nature. Additionally, it was reported that when an interpreter was present, the consultation time was even shorter and lasted typically around 4 min. Participants felt that this was insufficient to generate the trust required to discuss intimate details or present complex health issues.

### Theme 4: language and cultural competence

Communication difficulties with service providers were identified a key barrier for asylum seekers, refugees seeking health and social services. Findings indicate that the majority of asylum seekers had little or no English skills. The next passages substantiate the need for interpreters:

Some of us or some of the asylum seekers get acceptance to stay here they don’t know the English language well or maybe they are afraid to [express themselves] about medical conditions, about the service.

Often, persons with minimal English are less able to or in some cases unable to seek medical attention if an interpreter is not present. For instance, this service provider emphasized that ‘without an interpreter sometimes, they cannot go to the doctors.’ Additionally, miscommunication between doctors and patients frequently occur even in the presence of interpreters (specifically, persons who are not proficient in using medical terminology). This can lead to inaccurate diagnoses and inappropriate treatments:

That’s a problem. They, the doctor has to understand. I know someone that went to the doctor who doesn’t speak English, not one word, and goes to the doctor and the doctor gives a tablet or gives something, but how, if he doesn’t understand where the pain is.

In terms of availability, many reported noted that interpreters were, largely, not available at GP offices and hospitals. When asked if interpreters were a necessity, the answer was always, ‘Yes, of course.’ And when asked if this service was ever available, the response was consistently, ‘No, never.’ One participant expressed experiencing difficulties when arranging for a surgical procedure at the hospital:

Yeah, sometime I need other interpreter, translator. When I went for operation, before operation, this is big problem. I need translator, something more.

Another participant highlighted an incident that could have potentially resulted in a fatal outcome. Translated excerpts from field notes described the individual arriving at the hospital and being left in the waiting room unconscious. The nature of the illness was not specified, however, the individual had expressed how having an interpreter present could have alleviated the severity of the incident:

One participant pointed out that she was once brought to the hospital where she wasn’t conscious, and was left alone, waiting. When they saw her, they told her she could have died. She said she wouldn’t have minded having an interpreter if that could have helped for her, but they never offered her this service [researcher field notes].

Findings also highlight that, beyond language, there was a need for a more culturally competent system. For example, there is a need to address cultural differences concerning symptomologies, diagnoses and medical terminologies. The following passage describes the confusion that arose during a medical consultation through information was provided using very complex medical terms:

Well there’s a lot of information there. So when you go to the doctors, they don’t go into a lot of detail when they examine you and sometimes, when you’re learning English, you don’t learn the medical terms to express yourself.

As noted previously, it is important that when hiring interpreters or when clients are accompanied by interpreters who are friends or family members, that these advocates are also competent in interpreting medical terminology. Misinterpreted medical material are frustrating for patients and may lead to devastating outcomes such as adverse drug effects, permanent disability or even death.

Another issue concerning interpreters involves confidentiality. Often, interpreters are brought in from the same community as the people who they are providing interpretation services for. Many refugee and asylum seeker service users are hesitant to reveal any personal information during the translation process for fear that their personal information might be disclosed to members of their community:

Yeah, yeah. Some people have reservations because err, if there is a Somali man or woman [as an interpreter] living with that patient in the same town and err, they feel sometimes that some information may go out and be gossip to other friends. Reservations, they have some reservations.

Furthermore, understanding body language and cultural relevance of the diagnosis as well as acknowledging cultural implications or associations of the ailment by service providers is also important. For instance, during a Somali focus group, one participant stated, ‘Doctors don’t take notice of us – they don’t read body language.’ Another participant described inconsistencies between GPs and the limited amount of time each GP had to integrate cultural understandings associated with the ailment:

They [GPs] have their own style of examining and there, err, there can be such patients that you cannot examine properly in the limited time the GP has his fixed intention of making 5 min, 10 min, he won’t have had much time to assess, to go in depth in cultural situation […]

The analysis revealed a strong need for more culturally and linguistically diverse health service providers (particularly persons who are representative of smaller ethnic communities i.e. Somali and Iraqi) in health and social care settings to provide culturally-safe^2^ care. For example, some Somali participants indicated that they did not relate to the concept of depression; rather in their culture depression is characterized as a ‘complete madness.’ Limited knowledge of various culturally-specific mental health conceptualizations could result in appropriate diagnoses and treatment for mental health conditions such as depression. Yet depression was found to hugely problematic during our research as indicated by this Iraqi participant.

For me to be out of work affects me psychologically and I’m starting for the first time to experience depression and I’m afraid to stay for another 1 year, because I can’t return back penniless and I might be in danger also so I’ve seen some people like me, they’ve maybe been here two more years than me so I’m just afraid the longer to stay the more I will be depressed. The more I will delay to get indefinite leave to remain, the more I will be depressed. I’m afraid of my health if I don’t see my family soon.

In relation to issues associated with health literacy^3^, the analysis further identified the need for culturally appropriate information on disease prevention and health promotion. Key health literacy challenges relate to: the inability to thoroughly explain health problems using basic English; short doctor-patient consultations and lack of cultural awareness and/or sensitivity to ethnic minority patients by health care professionals.

## Discussion

In this article, we highlight the primary needs and concerns of Somali and Iraqi asylum seekers, refugees and individuals without legal status when accessing health care, supports and services. Specifically, we strived to build on previous anti-racist and feminist research focusing on health and social services for ethnic minority populations [27], which highlight the need to redevelop strategies to support marginalized groups, especially those that are constituted as being ‘seldom heard’ from countries experiencing political upheaval.

The analysis reveals how macro- and micro-level intersections of accustomed societal beliefs, practices and norms, broad-level legislation and policy decisions, and health care and social services delivery methods have affected the health and health care experiences of forced migrants that reside in the UK. Experiential findings reveal how the process of ‘othering’ grounded in Phoenix’s [9] notion of, ‘pathologized presence, normalized absence’ generates multiple forms of oppression. Ideas behind this phrase serve well to exemplify the experiences of asylum seekers, refugees and those with uncertain legal statuses in health care settings. The ‘pathologized presence’, predominantly, of asylum seekers and refugees embedded in processes of UK’s health care service delivery methods are further questioned here, as these key aspects play a vital role in determining health outcomes.

For participants of this study, health and health care experiences were associated with extraordinary circumstances such as, war, and settlement. Such experiences are shaped by varied and combined social identities concerning ‘gender’, ‘class’, ‘race’, ‘age’ and ‘culture’ (among other social categories); often characterized as essentialized features of the ‘other’ that delineate ‘minoritization’ or ‘othering’ processes [7]. Research highlights how ‘minoritization processes,’ influencing the intersections between social identities, can hinder access to and delivery of health and social services to vulnerable groups [10, 28–33]. Similar findings were reported here; and the most influential mechanism directly impacting health and access to health and social services was legal status.

For example, despite increased needs, the UK government firmly enforced legislation that mandated health care providers to charge ‘failed asylum seekers’ for health care services [34]. Interestingly, similar legislation has been adopted in other Western nations [35, 36]. In Australia, new legislations that restrict refugees’ access to health care have resulted in detrimental health outcomes. Restricting access to health services have caused refugees to spend prolonged periods of time in various Australian communities without undergoing basic health screening. Consequently, concerns regarding the spread of communicable diseases [36] amongst forced migrants have heightened, which in turn, has had stigmatizing effects.

Findings further highlight the importance of considering the ‘place’ which asylum seekers, refugees and persons without legal status occupy in British society. While citizenship has been historically defined by its association with ‘borders’ or ‘boundaries’, citizens are individuals who are part of the nation state. Therefore, we must query where asylum seekers and other state less individuals are situated within society whilst they are not legally considered as citizens. The legal-political construct of citizenship is associated with the guarantee of basic human rights for its citizens [37]. However, since asylum seekers, refugees and persons without legal status do not hold the legal title of being a British Citizen, their human rights, whilst they reside within UK borders, are not guaranteed. It is therefore crucial to address sanctions that situate stateless persons in the UK (particularly those that have sought refuge from war-torn countries) in incredibly vulnerable positions.

In terms of health care, our findings suggest that language and cultural differences can hinder service provision. Researchers and practitioners have called for the development of new roles for health care staff (including ancillary workers), in particular, to consider exceptional life circumstances (e.g. war, separations, death, and others) and adaptation to the hosting country of diverse refugee groups. In addition, there is a need for health services to develop and improve language and information services, close links with community-based organizations, specialist mental health services and services for survivors of torture and organized violence, as well as targeted health promotion and training of health workers [38]. Other studies [24, 39] have highlighted that new roles and responsibilities have been assigned to refugee community-based organizations, however, these services are often not properly equipped financially and structurally to cope with the demand.

With respect to health research focusing on different populations of asylum seekers, refugees and persons without legal status, in general, there is the problematic assumption that health issues are individually located and experienced. Such an approach operates within the premise that the asylum seeker or refugee carries with them the problem and the responsibility to solve that problem. This frame of mind holds individualized and westernized constructions of health, which seek to locate solutions and place responsibilities on the individual. Often, these are persons in extremely vulnerable positions who are denied the means to take control of their health and of their life circumstances. Consequently, one of the main criticisms of individual, clinically based services, particularly in the area of mental health, is that they are neither pragmatically possible nor culturally appropriate [40]. Therefore, we stress the need for future health services research to engage in more community level interventions focusing on services that are more culturally congruent with the communities’ own health constructions. Lastly, it is recommended that programmatic efforts and strategies in cultural safety be employed particularly within primary health care. Envisioning culture, ethnicity and race as fixed concepts can perpetuate stereotypical depictions of lifestyle and behaviour, which can incorporate culture-blaming ideologies. Cultural safety requires genuine efforts to understanding cultural barriers through community engagement and working with members of the community to addressing these barriers [41]. It also forces considerations of the differential power dynamics that exist between service providers and the service users. Incorporating cultural safety principles in health care practices and research may shift health structures to resist dominant essentialist views of culturally and socially heterogeneous groups.

In terms of study limitations, these were associated with the essence of conducting participatory methods. Despite its socially-driven and equity-focused principles, participatory methods are often resource intensive and time consuming; particularly since the research is embedded within the community and gaining access to community members require dedicated time to build partnerships, demonstrate accountability and ultimately to develop trust. For the current research, an important step toward circumventing challenges associated with establishing trust was through active communication as language barriers were reduced via appropriate interpreters with strong community ties. However, despite having interpreters present, it was often difficult to interpret medical terminology since certain terms were non-existent or represented differently in other languages. Furthermore, due to the sensitive nature of our research topic, one issue relating to trust transpired because some interpreters were from the participants’ community and as a result there was apprehension that confidential discussions may filter back to community members. Sensitive topics often became difficult for various individuals. For example, one participant was taken to an acute mental health service by the researcher due to concern for self-harm. In this instance, the flexibility of the methods, the ability to delve into issues when individuals are willing and able to talk combined with the centering of seldom heard voices offset challenges. Overall, participatory methods are unique in that they are firmly grounded in principles of empowerment. This insightful methodological strength often supersedes its limitations.

## Conclusions

Our study aimed to deepen understandings of how health and health care experiences of Somali and Iraqi asylum seekers, refugees and persons without legal status in the UK are shaped by social and structural determinants of health. The analysis focused on exploring how ‘othering’ and ‘minoritizing’ processes created dynamics, which ‘pathologized’ the presence and ‘normalized’ the absence of these groups. Personal accounts explained how immigration systems and structures (such as transient legal statuses) prevented access to vital resources such employment, education, appropriate housing, health care and public funding. Past lives and current social situations, including resettlement, asylum-seeking processes, hostility, racism and social isolation were also predictors of health and well being.

In terms of primary health care access, findings highlight that issues associated with mistrust, long wait times and rushed doctor-patient consultations were challenges for acquiring sufficient health care treatments and supports. To improve confidentiality and trust, care must be taken when hiring interpreters, especially for groups situated in very particular social and legal circumstances. More importantly, our findings identify a crucial need to re-evaluate the current asylum seeking process in order to initiate the development of a more humane system by creating policies that provide more access to funding and resources for individuals without status.

## Reflexive thoughts

At the outset, we were all very excited about this project because relatively little research has captured the health and health care experiences of asylum seekers, refugees and persons without legal status who the participants describe as ‘being in limbo’. Through the application of a participatory research framework, we felt strongly that this was an opportunity to prioritize seldom heard voices from Manchester’s Somali and Iraqi community. Local partnerships consisting of community, voluntary and health organizations created much needed communication channels, which helped translate findings into recommendations and enabled enhanced uptake by decisions makers. Nevertheless, it is important for us as critical health scholars to problematize the fact that the project leads were white, middle-classed British women; positionalities that often come with notable access to resources and privileges as opposed to the participants’ positionalities of inequity. Interestingly, for this project, such social advantages frequently served as barriers particularly for accessing communities that have felt a certain mistrust of people that hold such privileges. Having a privileged outlook distanced more senior team members from the acquired data because they did not have similar migration histories and/or post arrival experiences to situate themselves in the new knowledge. This absence of knowledge became a disempowering part of the research process. Gradually, however, this became an affirmative catalyst for dismantling traditional power hierarchies and as a team we became less hierarchal and more collaborative together with community organizations and local stakeholders.

## Footnotes

1 Cultural safety is a concept that emerged from the field of nursing in New Zealand and firmly asserts a distancing from victim blaming through the dispelling of cultural stereotypes [33].

2 Section 4 support is part of UK’s Immigration and Asylum Act since 1999. For failed asylum seekers, Section 4 support may provide accommodation to those who have a temporary barrier to leaving the UK and who would otherwise be destitute [32].

3 Health literacy, more broadly, refers to accessing, understanding and using information to make health decisions and encompasses terms and actions such as health knowledge, health motivation and health activation [42].
